# Supplementary material for: Explicit size distributions of failure cascades redefine systemic risk on finite networks
Source: Sci Rep. 2018 May 2;8:6878. doi: 10.1038/s41598-018-25211-3 (PMC5932047; doi:10.1038/s41598-018-25211-3)
Supplement: Supplementary file 1 — Supplementary Materials [file 41598_2018_25211_MOESM1_ESM.pdf]

Supplementary Materials for Explicit size distributions of failure cascades redefine systemic risk on finite networks

Rebekka Burkholz,[1*] Hans J. Herrmann,[2 3] Frank Schweitzer,[1]

[1]Chair of Systems Design, ETH Zurich
Weinbergstrasse 56/58, CH-8092 Zurich, Switzerland
[2]Computational Physics for Engineering Materials, IfB, ETH Zurich
Wolfgang-Pauli-Strasse 27, CH-8093 Zurich, Switzerland
[3]Departamento de Física, Universidade Federal do Ceará
Campus do Pici 60451-970 Fortaleza, Ceará, Brazil
[*]To whom correspondence should be addressed; E-mail: rburkholz@ethz.ch.

Supporting Information (SI)

Derivation of the cascade size distribution for complete networks

We offer two different representations of the final cascade size distribution for complete networks: one that enumerates all possible combinations of failures that could have led to a cascade outcome, and one which can be deduced by the inclusion-exclusion principle and whose implementation requires less computational resources. Both representations rely on combinatorial arguments, which we explain next. First, we note that each non-failed node in the network is exactly in the same state. A node carries the load $\lambda[k]$, if k other nodes have failed already. If $\rho_{\triangle} = k/N$ is the final cascade outcome we know that all surviving $N-k$ nodes must have thresholds larger than this load $\lambda[k]$. The probability for this event is $\prod_{i=1}^{N-k} \mathbb{P}\left(\Theta_i > \lambda[k]\right) = \left(1 - F(\lambda[k])\right)^{N-k}$, since we assume that the thresholds are indepen-

dently distributed. There are $\binom{N}{k}$ different combinations of k surviving nodes out of the total N nodes. The remaining k nodes fail altogether with a probability p_k that needs to be determined. In summary, we can write the cascade distribution as:

$$P\left(\rho_{\triangle} = \frac{k}{N}\right) = \binom{N}{k} \left(1 - F\left(\lambda[k]\right)\right)^{N-k} p_k. \tag{1}$$

The probability p_k that all k of the remaining nodes fail is equivalent to the failure of all nodes in a network with k nodes, since non-failed nodes do not influence the amount of load that any of the k nodes carries. The load required to cause a node to fail is determined by the node's threshold, while the entire configuration of all thresholds defines the order of the nodes' failure. But not all threshold configurations lead to k failures. For instance, it is not enough that all k nodes have a threshold smaller than $\lambda[k]$. If each node has a threshold $\Theta > \lambda_0$, no node fails initially and the cascade cannot start. In each time step, enough failures need to occur to trigger new failures. This can be translated into a criterion that identifies the threshold configurations that lead to exactly k failures. Whenever a node's threshold lies in the interval $(\lambda[j-1], \lambda[j]]$, it fails after j nodes have failed before (with $j = 1, \ldots, N-1$). We denote the number of nodes with this property with m_j. All m_0 nodes with threshold in $(-\infty, \lambda[0]]$ fail initially, while all m_N nodes with thresholds in $(\lambda[N-1], \infty]$ never fail. This definition implies that all nodes with a threshold corresponding to m_j fail before or at the same time as the nodes corresponding to m_l, if $l > j$, since $\lambda[l] > \lambda[j]$. Each possible threshold configuration translates into a vector $\mathbf{m_N} = (m_0, \ldots, m_N)$. In the first cascade time step, all m_0 nodes fail. We require $m_0 > 0$, otherwise nothing happens. In the second time step, all nodes fail whose failure premises at least m_0 failed neighbors. This includes all nodes that correspond to m_1. In order to cause the failures of at least all nodes that belong to m_2 (or any $j > 2$), the total number of failures before (or at the same time) $m_0 + m_1$ needs to be at least 2. Continuing this argument, for each $j = 1, \ldots, k-1$ we require $m_j \geq j + 1 - \sum_{l=0}^{j-1} m_l$ and in total $\sum_{l=0}^{k-1} m_l = k$. We define the

set of all such causal configurations for a cascade of size k as

$$\mathbf{I_k} := \left\{\mathbf{m_{k-1}} \in \{0,\dots,k\}^k \Big| \sum_{l=0}^{j} m_l \geq j+1 (\forall j \in 0,\dots,k-2), \sum_{l=0}^{k-1} m_l = k\right\} \tag{2}$$

and sum the probabilities of all those configurations in order to calculate p_k. Since each node's threshold lies in the interval $(\lambda[j-1], \lambda[j]]$ with probability $F(\lambda[j]) - F(\lambda[j-1])$, we have

$$p_k = \sum_{\mathbf{m}\in\mathbf{I_k}} \frac{k!}{\prod_{j=0}^{k-1}(m_j!)} F(\lambda[0])^{m_0} \prod_{j=1}^{k-1} \left(F(\lambda[j]) - F(\lambda[j-1])\right)^{m_j}. \tag{3}$$

$$p_k = \sum_{l=0}^{k-1} (-1)^{k+l+1} \binom{k}{l} F(\lambda[l])^{k-l} p_l, \tag{4}$$

$l = 2,\dots,N$ with $p_0 = 1, p_1 = F(\lambda[0])$. A formal proof is given below.

Proof of Equation (4) for compete networks

We proof that the final cascade size distribution, $P(\rho_\triangle = k/N)$ on complete networks can be expressed as given in Eqn. (1) with the p_k as given in Eqn. (4) The probabilistic inclusion-exclusion principle (*1*) states that for events $A_1,\dots,A_n$ in an arbitrary probability space, one of these events occurs with probability:

$$\mathbb{P}\left(\bigcup_{j=1}^{n} A_j\right) = \sum_{j=1}^{n} (-1)^{j+1} \sum_{I_j} \mathbb{P}\left(\bigcap_{l\in I_j} A_p\right) \tag{5}$$

where $I_j = \{l_1,\dots,l_j\}$ is a set containing exactly j distinct indicees $l_i \in \{1,\dots,n\}$. In case that the probability $q_j = \mathbb{P}\left(\bigcap_{l\in I_j} A_p\right)$ only depends on the number j of events that are intersected, we have

$$\mathbb{P}\left(\bigcup_{j=1}^{n} A_j\right) = \sum_{j=1}^{n} (-1)^{j} \binom{n}{j} q_j, \tag{6}$$

as there exist $\binom{n}{j}$ different subsets of $\{1,\dots,n\}$ with j elements.

To proof Equation (4), we need to define appropriate events A_j so that $p_k = \mathbb{P}\left(\bigcup_{j=1}^{k} A_j\right)$ and $q_j = F\left(\lambda[k-j]\right)^j p_{k-j}$, where we associate index j with $j = k - l$. Then Equation (4) follows immediately from the stated principle. First, we recall that p_k is the probability that k fully connected nodes fail in a cascade process considering only these k nodes (so that none of the failures is additionally influenced by other nodes in the network). There are several threshold configurations that lead to this outcome. All of them are of the form that some $k-j$ nodes have failed first, while the remaining j nodes fail because of the $k-j$ failures before. Each A_j subsumes events where the same j specific nodes $i_1, \ldots, i_j$ have thresholds smaller than $\lambda[k-j]$ and the remaining $k-j$ nodes failed in a cascade without considering $i_1, \ldots, i_j$. Indeed, the union of all A_j coincides with all cascades of size k and each A_j occurs with probability q_j.

Derivation of the cascade size distribution for star networks

Next, we shift our focus to a finite star network consisting of N nodes. We only have two categories of nodes, the single hub in the center with high degree $d = N - 1$, and $N - 1$ leaf nodes, i.e., the nodes with degree 1. The central node has a prominent role, as it is the only node in the network that can further distribute accumulated load. Consequently, a cascade ends after maximally three time steps. Initial failures might happen at $t = 0$ before the central node fails (among other nodes) at $t = 1$, and its failure possibly causes neighboring nodes to fail at $t = 2$. If the center does not fail, initial failures cannot become further amplified and the cascade stops after $t = 0$. If the center fails initially, it can cause further failures at $t = 1$, but then the cascade stops. Because of the limited number of possibilities, we can take the history of the center into account when we formulate a closed-form solution for the distribution of the final cascade size $\rho_\star$ for general cascading processes.

We allow the central node's cdf F_c and load λ_c to be different from a leaf node's cdf F and

its load λ_r. As before, we assume that the load $\lambda_c[k]$ that the center carries only depends on the number of its failed neighbors k (and the network size N). A leaf node can fail initially with probability $F(\lambda_r[0])$ (with 0 neighboring failures) or, if the center has failed before, it can carry the load $\lambda_r[j,l]$, which depends on two additional variables that are determined by the history of the center. j denotes the number of failed leaf nodes that have distributed load to the center, before the center has failed. l indicates the number of nodes among which the accumulated load of the center is shared (when the center fails).

We simply add the probabilities for all different cases and obtain:

$$
\begin{aligned}
&P\left(\rho_\star = \frac{k}{N}\right) = \left(1 - F_c\left(\lambda_c[k]\right)\right) \binom{N-1}{k} F\left(\lambda_r[0]\right)^k \left(1 - F\left(\lambda_r[0]\right)\right)^{N-1-k} \\
&+ F_c(\lambda_c[0]) \binom{N-1}{k-1} \sum_{j=0}^{k-1} \binom{k-1}{j} F\left(\lambda_r[0]\right)^j \left(F\left(\lambda_r[j, N-1-j]\right) - F\left(\lambda_r[0]\right)\right)^{k-1-j} \\
&\times \left(1 - F\left(\lambda_r[j, N-1-j]\right)\right)^{N-k} + \binom{N-1}{k-1} \sum_{j=1}^{k-1} \binom{k-1}{j} F\left(\lambda_r[0]\right)^j \left(F_c\left(\lambda_c[j]\right) - F_c\left(\lambda[0]\right)\right) \\
&\times \left(F\left(\lambda_r[j, N-1-j]\right) - F\left(\lambda_r[0]\right)\right)^{k-1-j} \left(1 - F\left(\lambda_r[j, N-1-j]\right)\right)^{N-k}.
\end{aligned}
\tag{7}
$$

.The first summand considers the case when the center does not fail, while the second term adds the probability for the case when the center fails initially. Then, each of the other $k-1$ failures of leaves can either occur initially or because of a load distribution by the center. The size of this load might depend on the number of nodes l that fail initially together with the center, since these nodes cannot receive load after the failure of the center. The index j in the third term finally takes the events into account when j leaves have failed before the center.

References and Notes

1. Szpankowski W (2001) *Inclusion-Exclusion Principle*. (John Wiley & Sons, Inc.), pp. 49–72.
